# Development of a nanoparticle-assisted PCR (nanoPCR) assay for detection of mink enteritis virus (MEV) and genetic characterization of the *NS1* gene in four Chinese MEV strains

**DOI:** 10.1186/s12917-014-0312-6

**Published:** 2015-01-13

**Authors:** Jianke Wang, Yuening Cheng, Miao Zhang, Hang Zhao, Peng Lin, Li Yi, Mingwei Tong, Shipeng Cheng

**Affiliations:** State Key Laboratory for Molecular Biology of Special Economic Animals, Institute of Special Animal and Plant Sciences, Chinese Academy of Agricultural Sciences, Changchun, 130112 China; Jilin Teyan Biological Technology Company, Changchun, 130122 China

**Keywords:** Nanoparticle-assisted PCR, Mink enteritis virus, Nonstructural protein 1 gene, Genetic characterization, China type

## Abstract

**Background:**

Mink enteritis virus (MEV) causes mink viral enteritis, an acute and highly contagious disease whose symptoms include violent diarrhea, and which is characterized by high morbidity and mortality. Nanoparticle-assisted polymerase chain reaction (nanoPCR) is a recently developed technique for the rapid detection of bacterial and viral DNA. Here we describe a novel nanoPCR assay for the clinical detection and epidemiological characterization of MEV.

**Results:**

This assay is based upon primers specific for the conserved region of the MEV *NS1* gene, which encodes nonstructural protein 1. Under optimized conditions, the MEV nanoPCR assay had a detection limit of 8.75 × 10^1^ copies recombinant plasmids per reaction, compared with 8.75 × 10^3^ copies for conventional PCR analysis. Moreover, of 246 clinical mink samples collected from five provinces in North-Eastern China, 50.8% were scored MEV positive by our nanoPCR assay, compared with 32.5% for conventional PCR. Furthermore no cross reactivity was observed for the nanoPCR assay with respect to related viruses, including canine distemper virus (CDV) and Aleutian mink disease parvovirus (AMDV). Phylogenetic analysis of four Chinese wild type MEV isolates using the nanoPCR assay indicated that they belonged to a small MEV clade, named “China type”, in the MEV/FPLV cluster, and were closely clustered in the same location.

**Conclusions:**

Our results indicate that the MEV China type clade is currently circulating in domestic minks in China. We anticipate that the nanoPCR assay we have described here will be useful for the detection and epidemiological and pathological characterization of MEV.

**Electronic supplementary material:**

The online version of this article (doi:10.1186/s12917-014-0312-6) contains supplementary material, which is available to authorized users.

## Background

Mink enteritis virus (MEV), a member of the genus *Parvovirus* within the family *Parvoviridae*, and a subspecies of the feline parvovirus (FPV), is a single-stranded DNA virus with a genome length of approximately 5,094 nt [1-3]. The MEV genome contains two major open reading frames (ORFs), a 3’ half ORF encoding the nonstructural proteins NS1 and NS2, and a 5’ half ORF encoding the capsid proteins VP1 and VP2.

MEV causes mink viral enteritis, an acute and highly contagious disease whose symptoms include violent diarrhea, and which is characterized by high morbidity and mortality [4]. The initial description of the disease in Canadian minks in 1949 [5] was followed by the isolation and identification of the viral pathogen and development of a vaccine in 1952 [6]. The disease has since been reported in a number of other countries worldwide [2], including China [7], and poses a serious economic threat to the global mink fur farming industry [8].

Diagnosis of MEV constitutes an important measure for the control of the disease, and although a broad number of approaches have been adopted, they have their own disadvantages [4,9-16]. For example, although electron microscopy and virus isolation are highly specific and sensitive, they are often too time-consuming and expensive for routine clinical use. Moreover, the latex agglutination test is rapid but lacks specificity, and the haemagglutination inhibition test requires a continuous supply of fresh erythrocytes and is unsuitable for the detection of non-haemagglutinating MEV isolates [15].

Conventional polymerase chain reaction (PCR) has been widely used for the detection of MEV and other viruses [17] through amplification of the highly conserved *NS1* and *VP2* genes [13,14] and, together with restriction fragment length polymorphism (RFLP), has been used for differentiation of MEV vaccine and wild type strains [13]. In addition, real-time PCR have been developed for the detection and quantification of other parvoviruses, including canine [18-20], porcine [21-23], human B19 [24,25] and human 4 [26] parvoviruses.

Nanoparticle-assisted PCR (nanoPCR) [27] incorporates nanoparticles to improve the specificity and speed of the reaction, and has been successfully applied for the detection of pseudorabies virus [28], bacterial aerosols [29], porcine parvovirus [17] and porcine bocavirus [30]. Here we describe the development of a nanoPCR-based assay for rapid clinical detection and epidemiological characterization of MEV.

## Results

### Optimization of MEV nanoPCR assay conditions

**Figure 1:**
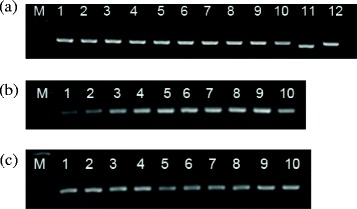
**Optimization of annealing temperature (a), primer concentration (b), and plasmid DNA concentration (c) for MEV nanoPCR.** Lane M: Low DNA Mass Ladder (Invitrogen, Carlsbad, USA); (a) lanes 1–12: The annealing temperatures were 48°C, 48.6°C, 49.4°C, 50.6°C, 52.2°C, 53.7°C, 54.9°C, 56.3°C, 57.8°C, 58.8°C, 59.5°C, and 60°C, respectively. (b) lanes 1–10: The primer volumes were 0.1 μL, 0.2 μL, 0.3 μL, 0.4 μL, 0.5 μL, 0.6 μL, 0.7 μL, 0.8 μL, 0.9 μL, and 1.0 μL, respectively. (c) lanes 1–10: The plasmid DNA volumes were 0.1 μL, 0.2 μL, 0.4 μL, 0.6 μL, 0.8 μL, 1.0 μL, 1.2 μL, 1.4 μL, 1.6 μL, and 1.8 μL, respectively.

Optimization of the nanoPCR assay encompassed adjustment of primer pairs, annealing temperature and the volumes of primer and plasmid DNA. Three primer pairs with fragment lengths of 194 bp, 163 bp and 389 bp, respectively, were compared, and based on gel quantification analysis by ImageJ 1.46r software, primer pair No. 1 (P1 and P2) was selected for use in conventional PCR and nanoPCR assays (data not shown). Band density was found to be optimal at an annealing temperature of 54.9°C, which was chosen for subsequent studies (Figure 1a). Using this annealing temperature, band density was found to be maximal at a primer volume of 0.6 μL (10 μmol/L) (Figure 1b) and a plasmid DNA volume of 1.0 μL (Figure 1c). Gel quantification analysis of all bands has been carried out using ImageJ 1.46r software (see Additional file 1).

Based on the results obtained with different annealing temperatures, primer volumes and plasmid DNA volumes for the MEV nanoPCR assay, an optimal 12 μL reaction volume was established, containing 6.0 μL of 2× nanobuffer, 0.6 μL each of the upstream and downstream primers (10 μmol/L), 1.0 μL of extracted DNA or standard plasmid, 0.2 μL of Taq DNA polymerase (5 U/μL) and ddH_2_O up to 12 μL. The reaction conditions were as follows: 3 min at 94°C, followed by 31 cycles at 94°C for 30 s, 54.9°C for 30 s and 72°C for 15 s, and a final elongation at 72°C for 10 min.

### Sensitivity of the MEV nanoPCR assay

**Figure 2:**
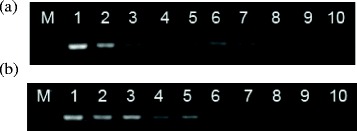
**Evaluation of the sensitivities of nanoPCR (a) and conventional PCR (b) for the detection of MEV** ***NS1*** **plasmid DNA.** Lane M: Low DNA Mass Ladder (Invitrogen, Carlsbad, USA); lanes 1–9: different MEV *NS1* plasmid DNA copies subjected to nanoPCR and conventional PCR (8.75 × 10^8^, 8.75 × 10^7^, 8.75 × 10^6^, 8.75 × 10^5^, 8.75 × 10^4^, 8.75 × 10^3^, 8.75 × 10^2^, 8.75 × 10^1^, and 8.75 × 10^0^ copies/μL, respectively); lane 10: blank.

Evaluation of the sensitivity of MEV nanoPCR assay indicated that the detection limit of the MEV nanoPCR assay (8.75 × 10^1^ copies/μL, Figure 2a) was 100-fold higher than that of conventional PCR analysis (8.75 × 10^3^ copies/μL, Figure 2b).

### Specificity of the MEV nanoPCR assay

**Figure 3:**
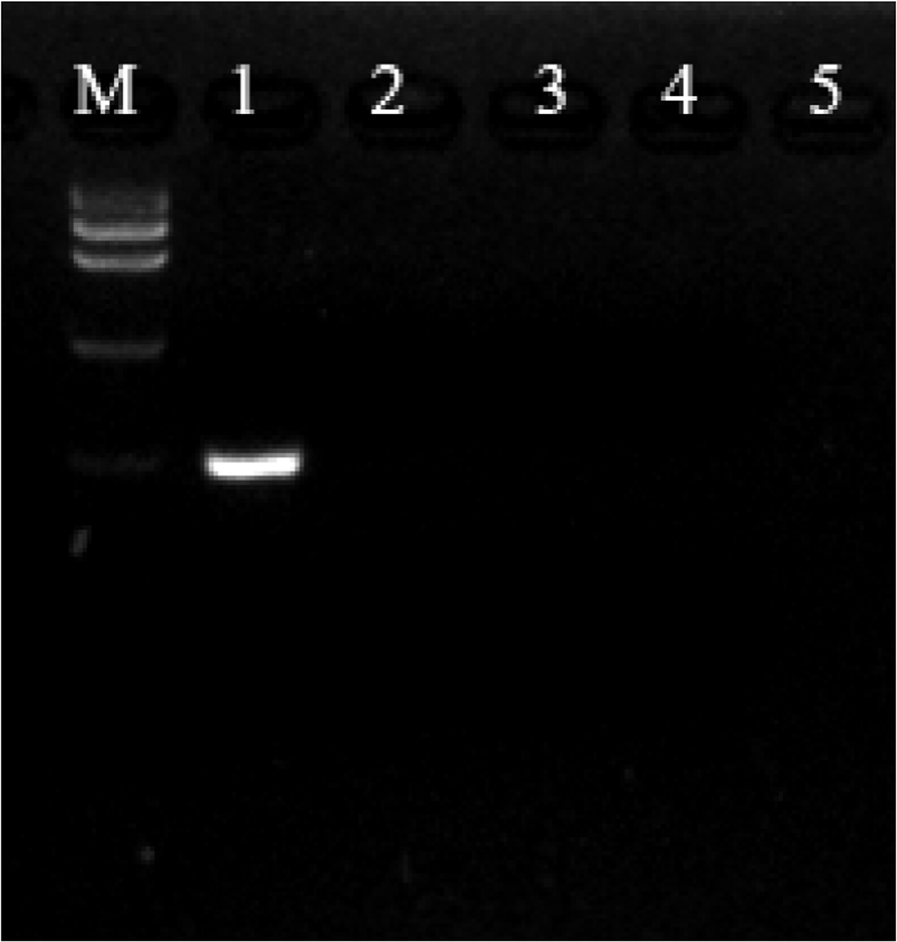
**Evaluation of the specificity of the MEV nanoPCR assay.** Lane M: Low DNA Mass Ladder (Invitrogen, Carlsbad, USA); lane 1: MEV genome as template; lane 2: cDNA of CDV genome as template, lane 3: AMDV genome as template, lane 4: DNA from fecal samples of healthy mink as template.

Agarose gel electrophoresis analysis indicated no cross reaction of the nanoPCR assay with CDV or AMDV DNAs, nor DNA extracted from the tissues of healthy minks, but was positive for MEV-infected minks (Figure 3).

### Diagnosis of MEV by nanoPCR assay

**Figure 4:**
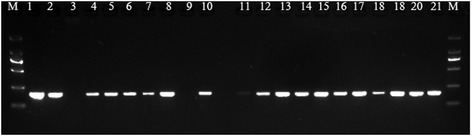
**Detection of MEV in clinical samples by nanoPCR assay.** Lane M: DL2000 DNA Maker (TaKaRa, Dalian, China); lane 1: MEV genome as template; lane 2: plasmid DNA as template; lanes 3: negtive control, lanes 4–21: DNA from clinical fecal samples as template.

Clinical samples were subjected simultaneously to MEV nanoPCR and conventional PCR. Eighty samples (32.5%) were positive for MEV by both nanoPCR and conventional PCR, and 121 samples (49.2%) were negative by both nanoPCR and conventional PCR. Forty five (34.3%) samples that were positive by nanoPCR were negative by conventional PCR, while no sample that was negative by nanoPCR was found to be positive by conventional PCR (Table 1). Compared with the conventional PCR, the relative specificity and sensitivity of nanoPCR were 72.9% (121/166) and 100% (80/80), respectively. The ten fecal samples from experimentally infected minks were positive for MEV by both nanoPCR and conventional PCR. Parts of clinical samples detection by MEV nanoPCR were shown in Figure 4.

**Table 1:** **Comparison of the sensitivity and specificity of nanoPCR and conventional PCR analysis for detection of MEV in fecal samples**

**nanoPCR**	**Conventional PCR**		
	**Positive**	**Negative**	**Total**
Positive	80	45	125
Negative	0	121	121
Total	80	166	246

Percentage of agreement: (80 + 121)/246 = 81.7%; relative sensitivity: 80/80 = 100%; relative specificity: 121/166 = 72.9%.

### DNA sequencing and phylogenetic analysis

**Figure 5:**
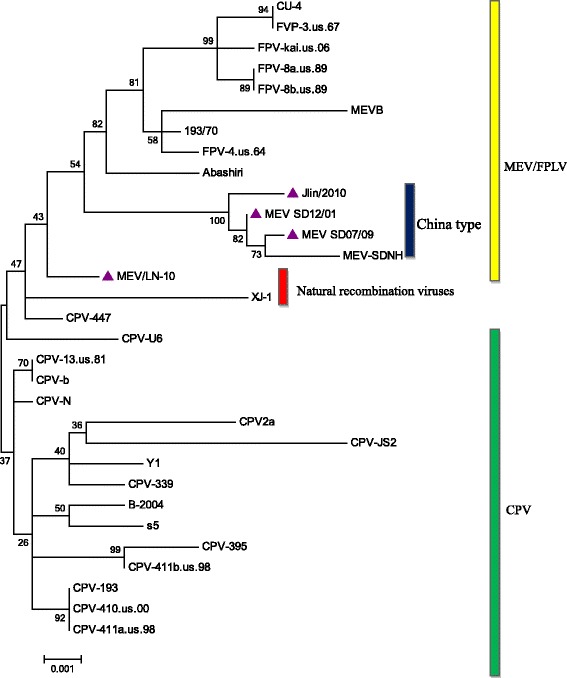
**Phylogenetic analysis of MEV with other carnivore parvoviruses based on** ***NS1*** **gene nucleotide sequences.** Nucleotide sequences were analyzed using the Maximum Likelihood method and Tamura-Nei model in MEGA6. Bootstrap values were calculated on 500 replicates. MEVs marked by solid triangles were isolated and preserved in our lab.

Sequence analysis indicated high similarity between the products obtained with the nanoPCR amplification of the *NS1* gene of MEV (the object sequences) and the reference sequence of MEV, indicating that the MEV nanoPCR is specific. A phylogenetic tree was constructed by the Maximum Likelihood method, and the robustness of the phylogenetic analysis was determined by bootstrap analysis with 500 replications (Figure 5). Analysis of this tree demonstrated that carnivore parvoviruses were divided into FPLV/MEV and CPV clusters. The MEV Jlin/2010, MEV-SDNH, MEV SD07/09, and MEV SD12/01 strains were classified into a small MEV clade, named the China type, in the FPLV/MEV cluster. Moreover, MEV/LN-10, a natural recombination virus between mink enteritis virus and canine parvovirus [31], was found to be more distant from the small China clade. In general, strains from the same province shared a common clade.

## Discussion

MEV is an important viral pathogen in the mink industry, causing high morbidity and mortality worldwide, and for which there are no effective treatments [32,33]. Accordingly, to improve epidemiological surveillance and prediction of the severity of MEV infection [4], we set out here to develop a simple and rapid diagnostic tool, targeting the conserved MEV *NS1* gene, for the detection and differentiation of MEV from other viruses.

A variety of methods currently exist for the detection of MEV, including the hemagglutination test and double antibody sandwich ELISA for the detection of MEV antigen [12], and the haemagglutination inhibition test, serum neutralisation test, and indirect ELISA for the detection of MEV antibodies [16]. These serological techniques, however, do not distinguish between vaccine or natural infection with wild-type virus as the cause of the antibody response. Moreover, although conventional PCR has been used to identify MEV infection [14,34], it is time-consuming and insensitive, and unsuitable for the detection of low viral loads in clinical samples. In addition, though LAMP assay is simple [4], it is readily subject to contamination.

The present study demonstrated that our nanoPCR assay is an effective and time-saving method for detecting MEV. This assay had 100-fold higher analytical sensitivity than conventional PCR, was specific for MEV, and exhibited no cross reactivity against other viruses. Of the 246 field samples in this study, 125 (50.8%) were positive for MEV when assayed by MEV nanoPCR, indicating the prevalence of MEV infection in China.

The results of our phylogenetic analysis, indicating that carnivore parvoviruses were divided into FPLV/MEV and CPV clusters, is similar to the results of a study based on VP2 gene sequences [31]. As shown in the phylogenetic tree, The strains MEV Jlin/2010, MEV-SDNH, MEV SD07/09, and MEV SD12/01 were classified into a samll MEV clade, named China type, in the FPLV/MEV cluster. The nucleotide divergence of the *NS1* gene between strains in the China type clade was between 0.1% to 0.5%, and that between China type and other carnivore parvoviruses was between 0.7% to 1.8%, with the exception of the natural recombination virus strain MEV LN-10 strain [31](data not shown). Specifically, the 357G, 516A, 570 T, 897G, 999A, and 1149G nucleotide residues in the *NS1* gene of the China type strains differed from those of all previously described carnivore parvovirus strains. All new mutations which did not result in amino acid residue replacement were synonymous substitutions.

In summary, we have developed a convenient nanoPCR method for the detection of MEV that is rapid, sensitive, and specific, and which detects both MEV field strains and vaccine strains. Compared with conventional PCR, this nanoPCR assay requires minimal laboratory facilities and is relatively simple and inexpensive to perform. Although only limited numbers of clinical samples were used in the present study, further studies will evaluate its performance in different laboratories and with a larger cohort.

## Conclusion

The nanoPCR assay developed in this study we have described here will be useful for the detection and epidemiological and pathological characterization of MEV. In addition, our results indicate that the MEV China type clade is currently circulating in domestic minks in China.

## Methods

### Viral strains and clinical samples

The viruses (MEV, CDV and AMDV) and 10 experimentally infected samples used in this study have been described in previous reports [4,35]. Animal experiments were approved by the Institute of Special Animal and Plant Sciences of CAAS, and animal experiments were performed in accordance with animal ethics guidelines and approved protocols. Fecal samples were obtained between 2007–2013 in Shandong, Hebei, Liaoning, Heilongjiang, and Jilin provinces, China, from 246 minks showing clinical and pathological signs of enteritis.

### Viral DNA/RNA extraction

Fecal samples were collected and stored by our group as previously described [4]. The MEVB or ADMV strain was propagated in the feline kidney F81 cell line in MEM medium. Virus particles were isolated from infected F81 cells when a cytopathic effect was visible about 96 hours after inoculation. Total DNA was extracted from fecal samples and from MEV- or ADMV-infected (positive control) and mock-infected (negative control) cell cultures using a DNA extraction kit (TaKaRa, Dalian, China) according to the manufacturer’s instructions. CDV RNA extraction and reverse transcription were performed as previously described [35].

### Primers and construction of recombinant plasmid DNA

A consensus MEV *NS1* gene sequence was obtained by aligning the genomes of different MEV isolates collected from publicly available sequence data (GenBank Accession Nos. D00765, FJ592174). Primers were designed using Primer Premier5.0 software (Molecular Biology Insights, Inc., Cascade, CO, USA) to amplify the full-length MEV *NS1* gene, with a predicted fragment length of 2,013 bp). The complete coding sequence of the MEV *NS1* gene was cloned into the plasmid vector pEASY-T1 (TransGen Biotech Company, Beijing, China) as the standard plasmid. The resulting pEASY-T1-MEV-*NS1* construct was amplified in E.coli DH5α, and the recombinant plasmid pEASY-T1-MEV-*NS1* was purified with the EasyPure Plasmid MiniPrep Kit (TransGen Biotech Company, Beijing, China) and quantified using a BioSpectrometer (Eppendorf, Hamburg, Germany) (8.75 × 10^10^ DNA copies/μL). Constructs were then confirmed by PCR and sequencing and kept at −20°C until use. An additional set of primers was designed to amplify a conserved portion of the *NS1* gene specific to MEV (GenBank accession number: FJ592174) (Table 2), with a predicted amplicon length of 194 bp.

**Table 2:** **NanoPCR and conventional PCR target gene and primers used for amplification of MEV**

**Primer name** ^**a**^	**Length (nt)**	**Genome position** ^**b**^	**Sequence (5′-3′)**	**Melting temperature (°C)**	**Product (bp)**
P1	20	1906-1925	ACAAGCGGCAAGCAATCCTC	54.9	194
P2	20	2080-2099	CTGCCTCTATTTCGGACCAT		
P3	23	151-173	CGCCATGTCTGGCAACCAGTATA	56	2013
P4	25	2139-2163	GGTTAATCCAAGTCGTCTCGAAAAT		

^a^P1 and P2 were used to amplify a portion of the *NS1* gene (194 bp). P3 and P4 were used to amplify the full-length MEV *NS1* gene (2,013 bp).

^b^The nucleotide positions of the nanoPCR and conventional PCR primers are according the genome sequence of mink enteritis virus strain MEVB (GenBank accession number FJ592174).

### Conventional PCR

MEV conventional PCR analysis was carried out using a primer set (P1 and P2, see Table 2) yielding a PCR product with a predicted length of 194 bp. PCR was carried out in a 20 μL reaction volume containing 1 μL extracted DNA or standard plasmid, 10 μL of 2× *EasyTaq* PCR SuperMix containing *EasyTaq* DNA polymerase, deoxynucleoside triphosphate (dNTP) and buffer (TransGen Biotech Company, Beijing, China), 7 μL ddH_2_O, and 1 μL of each of primers P1 and P2 (10 μM). The amplification regime was 5 min at 94°C followed by 31 cycles of 94°C for 30 s, 54°C for 30 s, and 72°C for 30 s, with a final elongation for 5 min at 72°C. PCR was carried out in a Life Express Thermal Cycler (HANGZHOU BIOER TECHNOLOGY CO., LTD, China). PCR products were subjected to electrophoresis on a 2% agarose gel.

### Optimization of MEV nanoPCR assay conditions

Optimization of the annealing temperature, plasmid DNA volume and primer volume for the MEV nanoPCR assay was carried out using the same primer pair as in conventional PCR for the MEV nanoPCR assay. Annealing temperatures in the Life Express Thermal Cycler ranged from 48°C to 60°C, the plasmid DNA volumes ranged from 0.1 to 1.8 μL, and the primer volumes ranged from 0.1 to 1.0 μL in increments of 0.1 μL. Products were visualized on 2% agarose gels at a voltage of 250 V for 15 min. The nanoPCR Kit (NPK02) was purchased from GREDBIO (Weihai, China). Gel quantification analysis of all bands was carried out using ImageJ 1.46r software (National Institutes of Health, Bethesda, MA, USA).

### Sensitivity of MEV nanoPCR assay

The limits of detection of for the MEV nanoPCR assay detection were compared with conventional PCR using a 10-fold dilution series of the pEASY-T1-MEV-*NS1* plasmid (ranging from 8.75 × 10^8^ to 8.75 × 10^0^ copies/μL), and using ddH_2_O was used as the negative control. PCR products were subjected to electrophoresis on a 2% agarose gel.

### Specificity of MEV nanoPCR assay

Cross-reaction of the MEV nanoPCR assay with AMDV DNA and CDV cDNA was evaluated using pEASY-T1-MEV-*NS1* as the positive control, and DNA extracted from fecal samples of healthy minks as the negative control. PCR products were subjected to electrophoresis on a 2% agarose gel.

### Detection of MEV in clinical samples

The sensitivity of the detection of MEV nanoPCR and conventional PCR assays was compared in clinical fecal samples from 246 minks in five provinces in North-Eastern China during the years 2007–2013. The location sources and the number of samples were as follows: Shandocng (122), Liaoning (31), Jilin (35), Heilongjiang (18), and Hebei (40) provinces. In addition, 10 fecal samples from experimentally infected animals were selected. Four of the positive products from the samples were sequenced.

### *NS1* gene sequencing and phylogenetic analysis

To determine the specificity of the MEV nanoPCR and the prevalence of MEV in China, the *NS1* genes from four MEVs (MEV Jlin/2010, MEV/LN-10, MEV SD07/09, and MEV SD12/01) detected by nanoPCR in the Jilin, Liaoning and Shandong province clinical samples were amplified, cloned, and sequenced as previously described [13,35]. The sequences of the full length 2,007 bp MEV *NS1* genes were assembled using the SeqMan and EditSeq functions of the DNAStar software package. Entire *NS1* gene sequences were aligned with the sequences of other carnivore parvovirus *NS1* genes collected from different locations worldwide (Table 3) and the consensus tree was edited in MEGA6. Phylogenetic analysis was performed using the Maximum Likelihood method, and setting the *p* distance algorithm of correction. Divergence was calculated by comparing sequence pairs in relation to the phylogeny reconstructed by MegAlign.

**Table 3:** **Nucleotide sequence accession numbers of MEV, CPV and FPLV isolates analyzed in this study**

**No.**	**Strains**	**Accession no.**	**Genetic type**	**Host**	**Submitted year**	**Origin**
1	Abashiri	D00765	MEV	mink	2007	Japan
2	MEVB	FJ592174	MEV	mink	2009	China
3	*MEV/LN-10*	*HQ694567*	*MEV*	*mink*	*2011*	*China*
4	*MEV SD12/01*	*KC713592*	*MEV*	*mink*	*2012*	*China*
5	MEV-SDNH	JX535284	MEV	mink	2013	China
6	*MEV SD07/09*	*KM099273*	*MEV*	*mink*	*2014*	*China*
7	CU-4	M38246	FPLV	feline	1996	USA
8	193/70	X55115	FPLV	feline	2005	USA
9	XJ-1	EF988660	FPLV	feline	2007	China
10	FPV-8a.us.89	EU659113	FPLV	feline	2008	USA
11	FPV-4.us.64	EU659112	FPLV	feline	2008	USA
12	FPV-3.us.67	EU659111	FPLV	feline	2008	USA
13	FPV-kai.us.06	EU659115	FPLV	feline	2008	USA
14	FPV-8b.us.89	EU659114	FPLV	feline	2008	USA
15	CPV-N	M19296	CPV-2	canine	1995	USA
16	CPV-b	M38245	CPV-2	canine	1996	USA
17	Y1	D26079	prototype CPV-2a	canine	2002	Japan
18	CPV2a	AJ564427	new CPV-2a	canine	2004	India
19	CPV-193	AY742932	new CPV-2b	canine	2005	USA
20	CPV-339	AY742933	new CPV-2a	canine	2005	New Zealand
21	CPV-447	AY742934	new CPV-2b	canine	2005	USA
22	CPV-U6	AY742935	new CPV-2a	canine	2005	Germany
23	CPV-395	AY742936	new CPV-2b	canine	2005	USA
24	B-2004	EF011664	new CPV-2a	canine	2006	China
25	CPV-13.us.81	EU659118	prototype CPV-2a	canine	2008	USA
26	CPV-410.us.00	EU659119	new CPV-2b	canine	2008	USA
27	CPV-411a.us.98	EU659120	new CPV-2b	canine	2008	USA
28	CPV-411b.us.98	EU659121	new CPV-2b	canine	2008	USA
29	CPV-JS2	KF676668	CPV-2a	canine	2013	China
30	s5	KF638400	CPV-2a	canine	2014	China

## Additional file

Additional file 1:
**Gel quantification analysis of all bands by ImageJ.**

## References

[1] Decaro N, Buonavoglia C (2012). Canine parvovirus–a review of epidemiological and diagnostic aspects, with emphasis on type 2c. Vet Microbiol.

[2] Steinel A, Parrish CR, Bloom ME, Truyen U (2001). Parvovirus infections in wild carnivores. J Wildl Dis.

[3] Kariatsumari T, Horiuchi M, Hama E, Yaguchi K, Ishigurio N, Goto H, Shinagawa M (1991). Construction and nucleotide sequence analysis of an infectious DNA clone of the autonomous parvovirus, mink enteritis virus. J Gen Virol.

[4] Wang J, Cheng S, Yi L, Cheng Y, Yang S, Xu H, Li Z, Shi X, Wu H, Yan X (2013). Detection of mink enteritis virus by loop-mediated isothermal amplification (LAMP). J Virol Methods.

[5] Schofield FW (1949). Virus enteritis in mink. N Am Vet.

[6] Wills CG (1952). Notes on infectious enteritis of mink and its relationship to feline enteritis. Can J Comp Med Vet Sci.

[7] Jiang TX, Pu HK, Wang L, Wang Y (1981). Preliminary report about mink viral enteritis disease. Fur animals.

[8] Hundt B, Best C, Schlawin N, Kassner H, Genzel Y, Reichl U (2007). Establishment of a mink enteritis vaccine production process in stirred-tank reactor and Wave Bioreactor microcarrier culture in 1–10 L scale. Vaccine.

[9] Shen DT, Ward AC, Gorham JR (1986). Detection of mink enteritis virus in mink feces, using enzyme-linked immunosorbent assay, hemagglutination, and electron microscopy. Am J Vet Res.

[10] Veijalainen PM, Neuvonen E, Niskanen A, Juokslahti T (1986). Latex agglutination test for detecting feline panleukopenia virus, canine parvovirus, and parvoviruses of fur animals. J Clin Microbiol.

[11] Uttenthal A, Larsen S, Lund E, Bloom ME, Storgard T, Alexandersen S (1990). Analysis of experimental mink enteritis virus infection in mink: in situ hybridization, serology, and histopathology. J Virol.

[12] Wang JK, Cheng SP, Yi L, Yang S, Luo B, Xu HL, Yan XJ, Wu H (2011). Establishment of double antibody sandwich ELISA for detection of mink enteritis virus. Chin Vet Sci.

[13] Wang JK, Cheng SP, Yang S, Yi L, Xu HL, Cheng YN, Shi XC, Wu H, Yan XJ (2012). Establishment of PCR-RFLP for differentiation of mink enteritis virus vaccine strain and wild strain. Chin Vet Sci.

[14] Zhang HL, Yan XJ, Chai XL, Wu W, Yi L, Luo GL, Tian HY, Shao XQ, Wang FX (2007). Establishment and application of PCR for detection of mink enteritis virus. Special Wild Econ Animal Plant Res.

[15] Rivera E, Sundquist B (1984). A non-haemagglutinating isolate of mink enteritis virus. Vet Microbiol.

[16] Chen T, Zhao JJ, Zhang HL, Chai XL, Yan XJ, Wu W, Qian AD (2009). Prokaryotic expression of mink enteritis virus VP2 gene and establishment of indirect ELISA. Chin J Prev Vet Med.

[17] Cui Y, Wang Z, Ma X, Liu J, Cui S (2014). A sensitive and specific nanoparticle-assisted PCR assay for rapid detection of porcine parvovirus. Lett Appl Microbiol.

[18] Elia G, Cavalli A, Desario C, Lorusso E, Lucente MS, Decaro N, Martella V, Buonavoglia C (2007). Detection of infectious canine parvovirus type 2 by mRNA real-time RT-PCR. J Virol Methods.

[19] Kumar M, Nandi S (2010). Development of a SYBR Green based real-time PCR assay for detection and quantitation of canine parvovirus in faecal samples. J Virol Methods.

[20] Mech LD, Almberg ES, Smith D, Goyal S, Singer RS (2012). Use of real-time PCR to detect canine parvovirus in feces of free-ranging wolves. J Wildl Dis.

[21] Chen HY, Li XK, Cui BA, Wei ZY, Li XS, Wang YB, Zhao L, Wang ZY (2009). A TaqMan-based real-time polymerase chain reaction for the detection of porcine parvovirus. J Virol Methods.

[22] Perez LJ, Perera CL, Frias MT, Nunez JI, Ganges L, de Arce HD (2012). A multiple SYBR Green I-based real-time PCR system for the simultaneous detection of porcine circovirus type 2, porcine parvovirus, pseudorabies virus and Torque teno sus virus 1 and 2 in pigs. J Virol Methods.

[23] Song C, Zhu C, Zhang C, Cui S (2010). Detection of porcine parvovirus using a taqman-based real-time pcr with primers and probe designed for the NS1 gene. Virol J.

[24] Koppelman MH, van Swieten P, Cuijpers HT (2011). Real-time polymerase chain reaction detection of parvovirus B19 DNA in blood donations using a commercial and an in-house assay. Transfusion.

[25] Zaki SA (2012). Detection of human parvovirus B19 in cancer patients using ELISA and real-time PCR. Indian J Med Microbiol.

[26] Vaisanen E, Lahtinen A, Eis-Hubinger AM, Lappalainen M, Hedman K, Soderlund-Venermo M (2014). A two-step real-time PCR assay for quantitation and genotyping of human parvovirus 4. J Virol Methods.

[27] Shen C, Zhang Z, Bagchi D, Bagchi M, Moriyama H, Shahidi F (2013). An Overview of Nanoparticle-Assisted Polymerase Chain Reaction Technology. Bio-Nanotechnology: A Revolution in Food, Biomedical and Health Sciences.

[28] Ma XJ, Cui YC, Qiu Z, Zhang BK, Cui SJ (2013). A nanoparticle-assisted PCR assay to improve the sensitivity for rapid detection and differentiation of wild-type pseudorabies virus and gene-deleted vaccine strains. J Virol Methods.

[29] Xu SY, Yao MS (2013). NanoPCR detection of bacterial aerosols. J Aerosol Sci.

[30] Wang X, Bai A, Zhang J, Kong M, Cui Y, Ma X, Ai X, Tang Q, Cui S (2014). A new nanoPCR molecular assay for detection of porcine bocavirus. J Virol Methods.

[31] Wang J, Cheng S, Yi L, Cheng Y, Yang S, Xu H, Zhao H, Yan X, Wu H (2012). Evidence for natural recombination between mink enteritis virus and canine parvovirus. Virol J.

[32] Sun JZ, Wang J, Yuan D, Wang S, Li Z, Yi B, Mao Y, Hou Q, Liu W (2013). Cellular microRNA miR-181b Inhibits Replication of Mink Enteritis Virus by Repression of Non-Structural Protein 1 Translation. PLoS One.

[33] Zhang QM, Wang YP, Ji Q, Gu JM, Liu SS, Feng X, Sun CJ, Li YY, Lei LC (2013). Selection of antiviral peptides against mink enteritis virus using a phage display peptide library. Curr Microbiol.

[34] Liu WQ, Fan QS, Jiang Y, Xia XZ, Huang G, Wang JG, Fang JB, Wang L (2001). Establishment of a commonly used PCR technique for detection of carnivore parvoviruses. Chin J Vet Sci.

[35] Yi L, Cheng S, Xu H, Wang J, Cheng Y, Yang S, Luo B (2012). Development of a combined canine distemper virus specific RT-PCR protocol for the differentiation of infected and vaccinated animals (DIVA) and genetic characterization of the hemagglutinin gene of seven Chinese strains demonstrated in dogs. J Virol Methods.

